# Use of Nanoparticles in Completion Fluids as Dual Effect Treatments for Well Stimulation and Clay Swelling Damage Inhibition: An Assessment of the Effect of Nanoparticle Chemical Nature

**DOI:** 10.3390/nano13030388

**Published:** 2023-01-18

**Authors:** Daniel López, Nicolas M. Chamat, Dahiana Galeano-Caro, Liliana Páramo, Diego Ramirez, David Jaramillo, Farid B. Cortés, Camilo A. Franco

**Affiliations:** 1 Grupo de Investigación en Fenómenos de Superficie-Michael Polanyi, Departamento de Procesos y Energía, Facultad de Minas, Universidad Nacional de Colombia—Sede Medellín, Medellin 050034, Colombia; 2 Parex Resources Colombia Ltd., Bogota 110111, Colombia

**Keywords:** completion nanofluid, nanoparticles, clay swelling, adsorption, interfacial tension, wettability

## Abstract

The objective of this study is to evaluate the role of nanoparticles with different chemical structures in completion fluids (CF) in providing a positive dual effect for well stimulation and clay swelling damage inhibition. Six types of commercial (C) or synthesized (S) nanoparticles have been incorporated into a commercial completion fluid. Doses varied between 100 and 500 mg·L^−1^. CF-nanoparticles were evaluated by fluid–fluid, fluid–nanoparticle, and fluid–rock interactions. The adsorption isotherms show different degrees of affinity, which impacts on the reduction of the interfacial tension between the CF and the reservoir fluids. Fluid–fluid interactions based on interfacial tension (IFT) measurements suggest that positively charged nanoparticles exhibit high IFT reductions. Based on contact angle measurements, fluid–rock interactions suggest that ZnO-S, SiO_2_-C, SiO_2_-S, and ZrO_2_ can adequately promote water–wet rock surfaces compared with other nanomaterials. According to the capillary number, ZnO-S and MgO-S have a higher capacity to reduce both interfacial and surface restrictions for crude oil production, suggesting that completion fluid with nanoparticles (NanoCF) can function as a stimulation agent. The clay swelling inhibition test in the presence of ZnO-S-CTAB and MgO-S-CTAB nanoparticles showed a 28.6% decrease in plastic viscosity (PV), indicating a reduction in clay swelling. The results indicate that a high-clay environment can meet the completion fluid’s requirements. They also indicate that the degree of clay swelling inhibition of the nanoparticles depends on their chemical nature and dosage. Finally, displacement tests revealed that CF with nanoparticles increased the oil linear displacement efficiency.

## 1. Introduction

Completion fluids (CF) are solid-free fluids commonly used in several field operations. Their purpose is to keep technical conditions and the bottom hole secure while operations are carried out [1,2]. A CF is typically composed of a brine of bromides or chlorides due to their high chemical compatibility with formation fluids and rock. However, any fluid with adequate density and flow conditions could be implemented as a completion fluid [1,2,3]. Drilling and/or completion operations in which the CF is used can involve extended direct contact with the reservoir fluids and rock. For this reason, their potential interactions with the formation should be considered during the CF formulation to avoid damage [4].

When the crude oil–brine–rock–CF interactions are not assessed, the CF can promote formation damage, which causes a reduction in reservoir porosity and permeability through multiple mechanisms. The mechanical mechanisms could be fines migration or phase trapping due to increased water saturation following CF leakage into porous media [5]. Chemical damage due to rock–fluid interaction, such as clay swelling or emulsion formation, may also exist in reservoirs [5,6,7,8]. The aqueous base CF system is prone to interact with the clay structure since the positive charge of H_2_O molecules is attracted by the negative superficial charge of clays; this causes the H_2_O molecules to fit within the crystallographic structure [9,10]. The presence of clay in sandstone reservoirs is common due to the geological formation process; its prevalence could be as high as 20% [11]. Although not all clays swell, they should be taken into account due to their potential to induce other formation damage, such as fine migration, when in contact with the CF’s aqueous filtrate [5,6].

The swelling process consists of two main stages related to reversible and irreversible processes. The reversible process is associated with crystal swelling, where the clay increases in volume [12]. In contrast, the irreversible process is related to osmotic swelling, which induces damage through fine migration. The damages can result in undesirable changes in the petrophysical properties of the reservoir and can generate risk to the integrity of the well when it is not ready to produce any substances [12,13]. Due to the high salt concentrations in the CF filtrate volume, other induced damage, such as wettability alterations from changes in the affinity of porous media, could be present during the completion operations [5,14]. These conditions reduce the productivity and technical and economic viability of the reservoirs [5].

The oil and gas industry typically faces clay swelling in sandstone reservoirs through stabilizers, such as inorganic salts (KCl, KNO_3_, NH_4_H_2_PO_4_, NH_4_Cl, and CaCl_2_), via cation exchange mechanisms and acid solutions (mud acid, retarded mud acid, and phosphonic-based HF) for clay dissolution [15,16]. Likewise, inorganic polymers (aluminum hydroxide, OH-Al, and ZrOCl_2_), organic salts (tetramethylammonium bicarbonate (TMAC) and choline chloride (CC)), and cationic/anionic organic polymers (poly quaternary ammonium (PQA) and hydrolyzed polyacrylamide (HPAM)) are commonly used for clay charge neutralization [17]. The main mechanism includes the cation exchange through the employment of inorganic salts, which is low-cost and HSE-effective (health, security, and environmental concerns), but requires high concentrations (2–7%) [17]. Consequently, innovative alternatives are needed without any risk regarding the alteration of fluid properties to better address the clay swelling issue.

Nanotechnology appears to be a solution for water-based fluids in clay swelling matters due to the simple synthesis of tailor-made nanoparticles to induce changes in the electrostatic interactions around the clay surface, which remediate the migration of the colloidal particles through the medium and hinder the swelling phenomena [18,19,20,21,22,23,24,25,26,27,28,29,30,31,32,33,34]. In this sense, several authors report using SiO_2_, MgO, ZnO, Al_2_O_3_, ZrO_2_, Fe_2_O_3_, and other nanoparticles due to their capacity to promote clay stabilization using electrostatic forces [4,17,20,35,36,37,38,39,40,41,42,43,44]. Huang and Clark [4] investigate the role of MgO nanoparticles in clay swelling in coreflooding tests using sand pack systems; they observe the pressure drop after the injection of 5% KCl solutions in the presence and absence of 0.4 wt% MgO nanoparticles, connecting the obtained results with the decrease in clay swelling and the clay stabilization processes. The authors also compared the behavior of commercial poly quaternary amine (PQA) with MgO nanoparticles in stabilizing bentonite and illite. The results suggest that even though both systems display the capacity to stabilize bentonite, MgO nanoparticles are better at stabilizing illite than PQA because of the increase in Van der Waals forces near the clay surface.

PEG-coated SiO_2_ nanoparticles at concentrations between 0.5–3.0 wt% were evaluated in the swelling of montmorillonite by Pham and Nguyen [37]. The results show a synergistic effect between nanomaterials and inorganic salts in reducing the swelling index. Pate et al. [20] evaluated the effectiveness of SiO_2_, Al_2_O_3_, ZnO, Fe_2_O_3_, and ZrO_2_ commercial nanoparticles in inhibiting natural bentonite clay swelling in deionized water and brine solutions of 4% KCl. The authors found that almost all the nanoparticles in deionized water exhibit reductions in the swelling index (SI) of over 40. When the nanoparticles were added to the brine solutions, all the nanoparticles reduced the SI by over 70% compared with brine solutions without nanomaterials. The authors evaluate the perdurability of the swelling inhibition by measuring SI after several washing processes and reveal that the presence of SiO_2_ nanoparticles mitigates clay swelling for 18 washes [20]. Finally, atomistic molecular dynamics simulations have been performed to study the interaction of SiO_2_ nanoparticles with Na-montmorillonite (MMT) clay platelets; this revealed the formation of electric double layers (EDL) around the MMT structure due to the accumulation of Na^+^, Ca^2+^, and Cl^-^ ions. The clay surface is modified by the insertion of ion-charged nanoparticles between platelets. The insertion promotes the control of swelling–shrinkage transitions of clays [18].

Oseh et al. [45] evaluated the (3–Aminopropyl) triethoxysilane polypropylene-nanosilica composite in a water-based drilling fluid for the inhibition of shale swelling and hydration. The swelling test showed a swelling of only 29% compared to the base system (62%). This result showed a better performance of the new system evaluated in comparison to the conventional systems of KCl and KCl+HPAM, which showed swelling of 42 and 33%, respectively [46]. Muhammad et al. found in their literature review that the presence of nanoparticles and surfactants in water-based drilling fluids reduce clay swelling. They also found that the inhibition is governed by ionic bonds, hydrogen bonds, and clay surface coatings; therefore, the chemical nature of the nanoparticles and surfactants would have a significant impact on the effectiveness of the inhibition.

While some of the developed nanoparticles for clay swelling inhibition have been incorporated into water-based drilling fluids [38,39,40,41,42,43,44], no study has focused on the addition of nanomaterials in CF systems for clay swelling issues. Only one study reports the use of nanoparticles in CF, focusing on modifying the capillary number (Nc) for the purpose of application in heavy crude oil reservoirs [30]. This study employed six different types of silica nanoparticles with different chemical properties to stimulate wells from the start of their productive lives. Nanoparticle behavior assessments were conducted by adsorption isotherms, interfacial tension measurements (IFT), and wettability alteration; the most suitable nanomaterial was evaluated using the coreflooding test [30]. The results show that the nanoparticles reduce surfactant adsorption by 75%, promote interfacial tension reductions of up to 76%, and enhance the wettability alteration of oil-wet to mixed-wet rock surfaces, leading to a 37% increase in oil effective permeability and increasing oil recovery by 3% [30]. Due to the poor knowledge of nanoparticle behavior when implemented in CF, nanoparticle screening must be assessed to identify their interactions with the rock surface and the reservoir fluids while evaluating their effectiveness in clay swelling inhibition.

The main objective of this manuscript is to evaluate the ability of metal oxide na-noparticles, such as SiO_2_, ZrO_2_, Al_2_O_3_, ZnO, and MgO, in CF to stimulate the well and inhibit clay swelling during completion operations. For this purpose, the adsorption of surfactant onto nanoparticles was carried out to determine the reduction in the adsorption of surfactant on the rock surface. In addition, IFT and contact angle measurements were used to estimate the capillary number (Nc). The inhibition of clay swelling was evaluated based on the study proposed by Barast et al. [47]. Considering the swelling inhibitory effect of hexadecyltrimethylammonium bromide (CTAB) [48,49,50,51], the best nanoparticles were selected and functionalized with this compound to improve the nanoparticles performance. Finally, the nanomaterial leading to higher Nc values and reduced clay swelling was evaluated in a core flood test to estimate the CF oil linear displacement efficiency curves in the presence and absence of the selected nanoparticle. This study is the first investigation aimed at developing dual-purpose nanomaterials to improve well completion operations by examining fluid–liquid–rock interactions aimed at reducing formation-induced damage and improving oil productivity.

## 2. Methodology

### 2.1. Materials

The effect of nanoparticles on commercial CF was studied by evaluating commercial and in-house synthesized nanomaterials. Commercial nanoparticles were labeled as X-C, where X is the nanoparticle identity. Otherwise, synthesized nanoparticles were labeled as X-S. Commercial oxide nanoparticles of ZrO_2_ (ZrO_2_-C) were provided by Nanostructured & Amorphous Materials (Katy, TX, USA). Additionally, Al_2_O_3_ (Al_2_O_3_-C) and SiO_2_ (SiO_2_-C) were provided by Petroraza S.A.S. (Medellín, Antioquia, Colombia). For the synthesis of SiO_2_-S, tetraethyl orthosilicate (TEOS > 99% Sigma-Aldrich, St. Louis, MO, USA) was employed as an organometallic precursor, ammonium hydroxide (NH_4_OH, 30%, J.T. Baker, Allentown, PA, USA) at 30 vol% as a catalyst, and ethanol (99.9%, Barecelona, Panreac, Spain) as a solvent. Furthermore, magnesium nitrate (MgNO_3_·6H_2_O) and zinc nitrate (Zn(NO_3_)_2_·6H_2_O) were provided by Panreac AppliChem (Castellar del Vallés, Barcelona, Spain). They were used as the nanoparticle precursors for MgO-S and ZnO-S, respectively. Sodium hydroxide (NaOH, >99% in volume fraction) obtained from Panreac AppliChem (Castellar del Vallés, Barcelona, Spain) was used during both synthesis procedures. Furthermore, n-heptane (99%, Sigma-Aldrich, St. Louis, MO, USA) was used in the rock samples’ aging. Potassium bromide (Pike Technologies, Fitchburg, WI, United States) was used for Fourier transform infrared (FTIR) analysis.

In the current study, an intermediate crude oil from the Magdalena Medio (MM) basin with a 28.9° API was used. The SARA analysis shows 53.35% of saturates, 24.21% of aromatics, 20.64% of resins, and 1.8% of asphaltenes.

The CF used was provided by a Colombian oil company and consisted of a water-base CF with a KCl concentration of 0.05 Kg·L^−1^, biocide concentration of 0.08 Kg·L^−1^, and a commercial surfactant concentration of 0.17 Kg·L^−1^. The properties of the formation brine and the commercial surfactant used are presented in Table 1. Additionally, according to the hydrophilic–lipophilic balance (HLB) = 14.02, the surfactant could be cataloged as a hydrophilic surfactant with a critical micelle concentration (CMC) of 2685.9 mg·L^−1^. CF and prepared completion fluid with nanoparticles systems (NanoCF) were employed in the static and dynamic tests.

An outcrop collection and characterization campaign were performed in the Magdalena Medio Basin to obtain representative core samples for the current study. Based on the mineralogy and petrophysical properties of the reservoir, the selected rock samples were aged in mixtures of 40%vol of MM crude oil and 60%vol n-heptane for 200 h at 80 °C [52,53]. The rock samples have been employed in evaluating the surfactant adsorption onto the rock surface and the wettability tests while selecting the most suitable NanoCF.

### 2.2. Nanoparticle Synthesis

#### 2.2.1. Synthesis of SiO_2_ Nanoparticles (SiO_2_-S)

The synthesis process of SiO_2_-S was carried out through the Stöber method; 312.5 mL of ethanol, 9 mL of TEOS, and 22.5 mL of NH_4_OH were added to a round-bottomed flask. The round-bottomed flask was attached to a condenser, and the mixture was stirred for 24 h at 60 °C. The solution was filtered with ethanol/water mixtures and dried at 70 °C [54,55].

#### 2.2.2. Synthesis of MgO Nanoparticles (MgO-S)

The MgO nanoparticle synthesis was completed using the Sol-Gel method proposed by Wahan et al. [56]. Aqueous solutions of 0.2 M MgNO_3_·6H_2_O were dissolved in 100 mL of deionized water. NaOH (1 M) was added dropwise into the prepared solutions of MgNO_3_·6H_2_O to achieve a pH of 12.5, which promotes the magnesium hydroxide precipitation. This mixture was continuously stirred at 500 RPM for 30 min. The residue was centrifuged for 15 min at 4500 RPM, cleaned with methanol to remove ionic impurities, and dried at 70 °C. The dried samples were calcinated at 400 °C for 2 h.

#### 2.2.3. Synthesis of ZnO Nanoparticles (ZnO-S)

The synthesis of ZnO-S was performed using the sol–gel method proposed by Pourrahimi et al. [57]. A 0.2 M 100 mL solution of Zn(NO_3_)_2_·H_2_O and a 0.5 M NaOH solution, both in deionized water, were employed. The NaOH solution was added dropwise into the Zn(NO_3_)_2_·H_2_O solution and continuously stirred until the pH reached 12. The mixture was stirred for 30 min at 500 RPM and separated through centrifugation for 20 min at 4500. The nanoparticles were washed three times with methanol and calcined in the muffle at 400 °C for 2 h.

#### 2.2.4. Nanoparticle Surface Modification

The selected nanomaterials were added to an aqueous solution of CTAB under a nanoparticle-to-CTAB weight ratio of 9:1. The mixtures were stirred at 350 rpm for 24 h at 25 °C. The solution was centrifuged at 3000 rpm for 15 min. Finally, the obtained nanoparticles were dried at 60 °C for 12 h.

#### 2.2.5. Nanoparticle Characterization

The identification of functional groups on the surfaces of the nanoparticles was performed through Fourier transform infrared spectroscopy (FTIR) using an IRAaffinity-1 spectrophotometer (Shimadzu, Kioto, Japan) from a range of 4500–400 cm^−1^ at a resolution of 4 cm^−1^ and using KBr as the blank. The samples were dried at 110 °C for 4 h and then mixed with KBr at a KBr:sample ratio of 80:20 [58,59,60]. The hydrodynamic diameter of the nanoparticles at 10 mg·L^−1^ in aqueous media was achieved using the dynamic light scattering (DLS) technique in a NanoPlus-3 device (Micromeritics, Norcross, GA, USA). For this technique, dry nanoparticles were added to an aqueous solution and sonicated for 2 h before the measurements were obtained. Point of zero charge (pH_PZC_) was achieved by preparing acid and alkaline aqueous solutions of the nanoparticles at 100 mg·L^−1^. Dry nanoparticles were added to aqueous solutions at different pHs, stirred at room temperature for 1 h, and then transferred to the zeta potential (ζ) cell. Zeta potential values for each system were obtained using a NanoPlus-3 device (Micromeritics, GA, USA), and pH_PZC_ was determined at a pH value where ζ is equal to 0 [61]. Nanoparticle surface total acidity was measured using the temperature-programmed desorption of NH_3_ (NH_3_-TPD) technique in a ChemBET TPR/TPD (Quantachrome Instruments, Boynton Beach, FL, USA) device [62,63]. Furthermore, the nanoparticle surface area (S_BET_) was calculated using the Brunauer–Emmett–Teller (BET) method and the adsorption isotherms of N_2_ at −196 °C using an Autosorb-1 (Quantachrome Instruments, USA) instrument. CTAB dosage on selected nanoparticles was determined using thermogravimetric analyses (TGA) in a Q50 analyzer (TA Instruments, Inc., New Castle, DE, USA). The samples were subjected to an air atmosphere, constant airflow of 100 mL·min^−1^, and a heating rate of 10 °C·min^−1^ from 20 to 800 °C. The weight differences between the raw and modified nanoparticles were used to estimate the surfactant dosage on the nanomaterial surface [64].

#### 2.2.6. Batch Adsorption Isotherms

The commercial surfactant adsorption onto the surface of the nanoparticles was carried out at 25 °C, at a solution volume-to-dry mass nanoparticle ratio of 0.1 L·g^−1^, and a fixed surfactant dosage ranging from 0 to 5000 mg·L^−1^. First, surfactant solutions in the presence of the nanomaterials were stirred for 12 h at 400 RPM to ensure the adsorption equilibrium. Second, the solutions were centrifuged for 45 min at 4500 RPM to promote the nanoparticle separation from the aqueous phase. The concentration of the non-adsorbed surfactant remaining in the solution was determined through a spectrophotometer UV-VIS (GENESIS 10S). A calibration curve was constructed between the absorbance of the aqueous phase vs. the surfactant concentration. The mass balance shown in Equation (1) reveals the adsorbed surfactant quantification:

(1) $$N_{ads}=\left(C_{i}-C_{E}\right)\frac{V}{M}$$

where $N_{ads}$ is the adsorbed amount (mg·g^−1^), $C_{i}$ (mg·L^−1^) is the initial concentration of surfactant, and $C_{E}$ (mg·L^−1^) is the concentration after the adsorptive equilibrium. Lastly, $\frac{V}{M}$ (L·g^−1^) is the ratio of solution volume per gram of adsorbent [65]. The experimental data were adjusted using the SLE (solid–liquid equilibrium) model to describe the adsorption isotherms based on the “chemical theory” [66]. The model is based on the following expression:

(2) $$C_{E}=\frac{\psi H}{1+K\psi }\exp\left(\frac{\psi }{N_{ads}}\right)$$

(3) $$\xi =\left(\frac{N_{m}\cdot N_{ads}}{N_{m}-N_{ads}}\right)$$

(4) $$\psi =\frac{-1+\sqrt{1+4K\xi }}{2K}$$

where $N_{m}$ (mg∙g^−1^) is the maximum adsorption value of surfactant onto nanoparticles under the studied range, $N_{ads}$ (mg∙g^−1^) is the amount of surfactant adsorbed onto the nanoparticle surface, and $C_{E}$ is the equilibrium surfactant concentration. The SLE parameter K (g∙g^−1^) is associated with the rapid surfactant self-association once the primary sites have been occupied. Additionally, H (mg∙g^−1^) is referred to as Henry’s law constant. The H is related to the affinity of the adsorbent for the adsorbate.

The root mean square error (*RMSE*%) was calculated to determine the fit of the model to the experimental values according to Equation (5) [67].

(5) $$RSME\%=100\sqrt{\overset{p}{\underset{i}{\sum }}\frac{{\left(X_{exp,i}-X_{cal,i}\right)}^{2}}{p}}$$

where $X_{exp,i}$ are the values of Nads for experimental data, $X_{cal,i}$ is the values of Nads for calculated data with the SLE model, and p is the number of data used.

#### 2.2.7. Wettability Test

**Figure 1:**
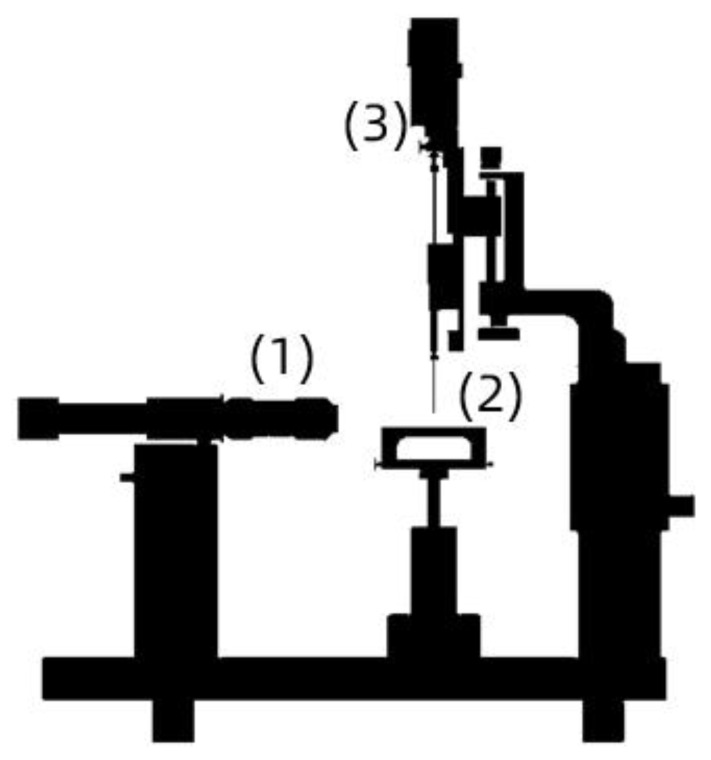
Schematic assembly of an optical tensiometer for contact angle measurements: (1) high-resolution camera, (2) sample support, and (3) droplet injection system.

Each outcrop sample was immersed in CF solutions in the presence of nanoparticles at dosages ranging from 50 to 500 mg·L^−1^ and then soaked for 48 h at 60 °C. The rock wettability before and after the sample immersion in CF/nanoCF solutions was verified by employing the sessile drop method in an Attention Theta optical tensiometer (Biolin Scientific AB, Västra Frölunda, Sweden) to estimate the water contact angle (WCA). At least three droplets of formation brine with a volume of 8 µL were added to the rock surface at different locations. The WCA was obtained at room temperature. Figure 1 shows the schematical arrangement for measuring the contact angle in an optical tensiometer.

#### 2.2.8. Interfacial Tension

Using the Wilhelmy plate method, the interfacial tension (IFT) value between MM crude oil and the prepared CF or NanoCF systems was developed in a force tensiometer Sigma 702 (Biolin Scientific, Espoo, Finland) for values higher than 1 mN m^−1^. For this method, a thin wire ring is placed below the interface between the CF or NanoCF and MM crude oil. The ring is then pulled up through the interface, and the pull force is estimated [68]. A spinning drop tensiometer SDT (Krüss GmbH, Hamburg, Germany) was used for IFT values under 1 mN·m^−1^. For this method, a MM crude oil droplet is placed in a capillary tube filled with CF or NanoCF. Next, the whole setup is rotated at high speeds. The centrifugal force during rotation promotes drop deformation. The droplet deformation allows the determination of the IFT value between immiscible phases. The IFT value is measured using Vonnegut’s equation [69], described as:

(6) $$\sigma =\frac{1}{4}\omega^{2}\left(\delta_{w}-\delta_{o}\right)r^{3}$$

where $\delta_{w}$ (g·L^−1^) is the brine density, $\delta_{o}$ is the crude oil density, r (mm) is the radius of the crude oil drop, ω (rpm) is the angular velocity, and σ (mN·m^−1^) corresponds to the IFT value between the phases. The IFT value was measured at room temperature for both devices.

#### 2.2.9. Capillary Number

The capillary number (Nc) indicates the viscous forces related to the CF or NanoCF viscosity, the fluid velocity, and the interface forces that occur when the CF or NanoCF make contact with the MM crude oil and the rock surface [50,70,71]. This dimensionless quantity is used to obtain information about how the nanoparticle affects the main mechanism involving oil mobilization when the CF interacts with the reservoir fluids and rock surface. The obtained WCA and IFT values were used as input variables for the estimation of the Nc using the following equation [30,72]:

(7) $$N_{c}=\frac{\mu V}{\sigma \cos\left(\theta \right)}$$

where μ is the displacement fluid viscosity (P), V is the average fluid velocity (cm·s^−1^), σ is the interfacial tension (mN·m^−1^), and θ is the contact angle formed between the surface rock and the displacement fluid [73].

#### 2.2.10. Clay Swelling Inhibition

The clay swelling mechanism was evaluated through the methodology proposed by Barast et al. [47]. Natural bentonite at 0.09 Kg·L^−1^ was added to CF/NanoCF systems. The rheology of the prepared solutions was measured using a rotational viscometer (Fann, Houston, TX, United States), with velocity varying from 3 to 600 RPM at 25 °C. The plastic viscosity (PV) of the CF or NanoCF muds in the presence of natural bentonite was used as the response variable to determine the clay swelling inhibition. This means that the PV of natural bentonite muds using deionized water was used as the blank. The PV was obtained as follows:

(8) $$\mathrm{PV}=\theta_{600}+\theta_{300}$$

where PV is the plastic viscosity (cP), and $\theta_{300}$ and $\theta_{600}$ are the Fann values at 300 and 600 RPM, respectively.

#### 2.2.11. Dynamic Test

An outcrop rock sample obtained from the MM basin was used in the following test. The piece was cleaned with a mixture of methanol/toluene and dried at 120 °C for 18 h before it was used. Table 2 shows the petrophysical properties of the rock sample.

**Figure 2:**
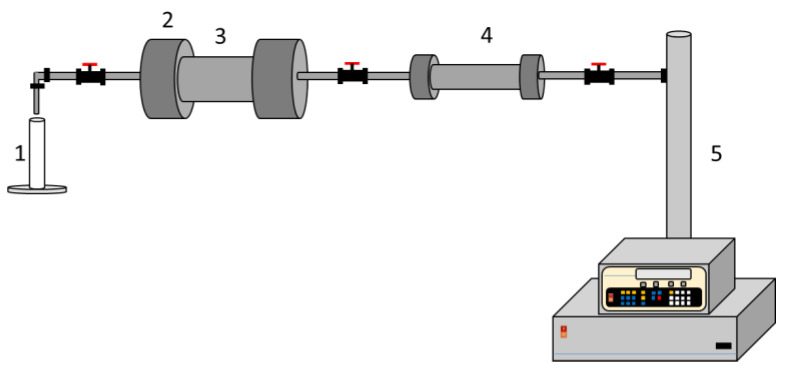
Experimental setup for the coreflooding tests: (1) test tube for sampling, (2) sample holder, (3) rock sample core, (4) cylinder, (5) syringe pump.

A coreflooding test was conducted at room temperature and pressure. The procedure was divided into three stages: (i) Construction of the oil linear displacement efficiency baseline, (ii) CF or NanoCF soaking in the porous media for 48 h, and (iii) construction of the oil linear displacement efficiency curves after the sandstone core makes contact with CF or NanoCF. The experimental setup is shown in Figure 2. First, 10 pore volume of the production brine was injected at 0.2 mL∙min^−1^. Then, 10 pore volume of MM crude oil was injected, followed by 10 pore volume of the production brine to construct the baseline oil linear displacement efficiency curves. Afterward, 10 pore volume of MM crude oil was injected to prepare the porous media for the CF or NanoCF injection. Finally, 10 pore volume of the formation brine was injected to construct the linear displacement efficiency curves, 0.5 pore volume of CF/NanoCF was injected, and the porous media was soaked for 48 h.

## 3. Results

### 3.1. Nanoparticle Characterization

**Figure 3:**
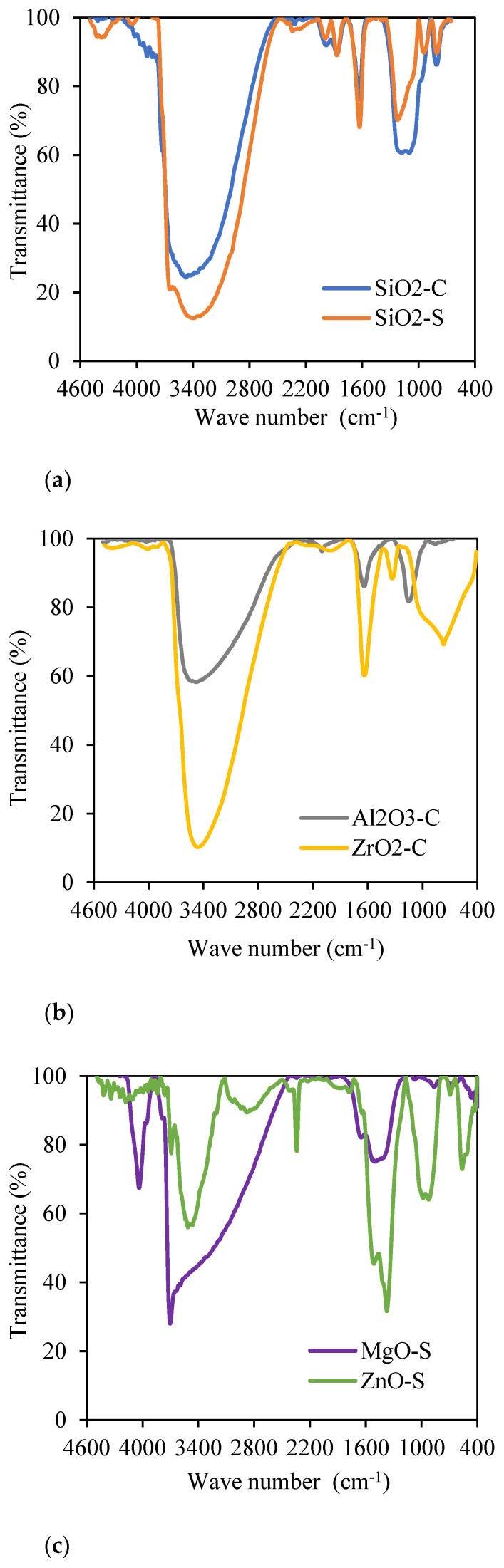
FTIR analysis for: (**a**) SiO_2_-C (blue curve) and SiO_2_-S (orange curve); (**b**) Al_2_O_3_-C (grey curve) and ZrO_2_-C (yellow curve); (**c**) MgO-S (purple curve) and ZnO-S (green curve).

Fourier transform infrared spectroscopy (FTIR) was carried out to identify functional groups, and the results are shown in Figure 3. One noteworthy result is the water molecules interacting with hydroxyl groups on the surface of all assessment nanoparticles [56,74,75]. Figure 3a exhibits the IR spectrum for SiO_2_-C and SiO_2_-S nanoparticles, where the stretching of Si-OH functional groups is present in wavelengths 3500 cm^−1^ to 3400 cm^−1^ and in the range from 1650 cm^−1^ to 1600 cm^−1^ [74,76]. The Si-O covalent bonds were observed in the range from 1300 to 1000 cm^−1^ [77,78], and the Si-O-Si functional groups are present near the frequency of 800 cm^−1^ [79]. The intensities of the bands corresponding to the functional groups Si-OH and Si-O-Si differ due to the content of these groups on the surfaces of the nanoparticles [80]. While there is a higher proportion of Si-OH groups in SiO_2_-S nanoparticles due to their lower transmittance at 3500-3400 cm^−1^, the SiO_2_-C exhibits a higher proportion of Si-O-Si groups near 1200 cm^−1^. This behavior is expected according to the research by Montes et al. [81]. It confirms the greater %O/%Si proportion of SiO_2_-C regarding the SiO_2_-S. This proportion affects their textural properties, surface chemical properties, and, consequently, their performance in lab tests.

Figure 3b shows the IR spectrum for ZrO_2_-C and Al_2_O_3_-C, where vibrational bands from 3500 cm^−1^ to 1650 cm^−1^ are evidenced by the water molecules adsorbed onto the nanoparticle’s surface [75]. The functional group Al-O of Al_2_O_3_-C nanoparticles is exhibited in vibrational bands of approximately 1000 cm^−1^, and the hydroxyl group Al-OH is present in the small peak between the frequencies of 1650 and 1600 cm^−1^ [82]. The interaction of functional groups Zr-OH and Zr-O-Zr on the nanoparticle’s surface are represented at 1350 and 700 cm^−1^, respectively [83]. Figure 3c presents IR spectra for synthesized ZnO-S and MgO-S, in which hydroxyl groups (Zn-OH and Mg-OH, respectively) vibrate on both nanoparticle surfaces. For ZnO-S case, this is represented in bands near 3500 cm^−1^ and is associated with their hygroscopic chemical nature [84]. For MgO-S nanoparticles, functional groups have two wider wavelength spectra from 4000 to 3500 cm^−1^ and from 1500 to 1400 cm^−1^; they are associated with interactions with water molecules [56]. Additionally, in the IR spectrum for ZnO-S, Zn-O functional groups vibrate in bands from 440 to 430 cm^−1^ [82], and the presence of MgO-S is confirmed for Mg-O in the wavelengths 800–500 cm^−1^ [85].

The point of zero charge (pH_pzc_), surface areas (S_BET_), and hydrodynamic diameter are shown in Table 3. The results show that the hydrodynamic diameters of nanoparticles are between 11 and 150 nm and follow the order: SiO_2_-C < ZrO_2_-C < SiO_2_-S < MgO-S < ZnO-S < Al_2_O_3_-C.

As expected, they maintain an inverse relation with the surface area for the mass unit. The decrease is in the following order: SiO_2_-C > MgO-S > ZrO_2_-C > Al_2_O_3_-C > SiO_2_-S > ZnO-S; the smallest value of SiO_2_-C (11.8 nm) is due to its highest surface area for the mass unit (380 m^2^∙g^−1^). In the case of the pH_pzc_ value, the highest pH_pzc_ of all assessed nanoparticles corresponds to MgO-S (pH_pzc_ = 11.6) and ZnO-S (pH_pzc_ = 8.1); this suggests that the nanoparticles display a positive surface charge. Nanoparticles with a pH_pzc_ below 7, such as ZrO_2_-C, SiO_2_-S, and SiO_2_-C, will have a negative charge on their surface and inverse behavior; these factors prevent interaction with the charges of fines clays for formation damage associated with increasing water saturation at the reservoir [86]. Finally, for the Al_2_O_3_-C nanoparticles, which present a pH_pzc_ of 6.3, it is expected that the interaction with the fluid is present in the aggregation and instability phenomenon [87].

### 3.2. Adsorption Isotherms

**Figure 4:**
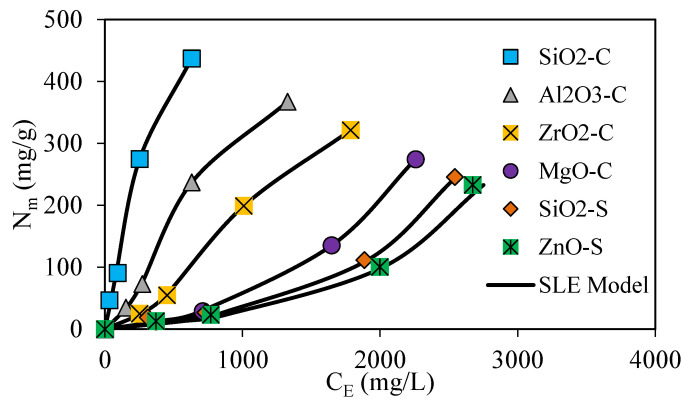
Adsorption isotherms at 25 °C and fixed surfactant dosage ranging from 0 to 5000 mg·L^−1^ for SiO_2_-C (blue dots), Al_2_O_3_-C (grey dots), ZrO_2_-C (yellow dots), MgO-C (grey dots), SiO_2_-S (orange dots), and ZnO-S (green dots), along with SLE model fitting (continuous black line).

Batch adsorption isotherms were performed to determine the interactions between the surfactant in the CF formulation and the nanoparticles. The results were analyzed with the SLE model displayed in Figure 4.

The isotherm results enable the quantification of the surfactant amount that is absorbed onto the nanoparticle’s surface instead of directly positioned onto the rock surface. The results provide more insight into the phenomenon that leads to the changes in petrophysical properties and fluids’ interfacial tension dynamics at the reservoir with and without the nanoparticle implementation [30,88,89,90,91]. Based on the IUPAC classification and the nanoparticle’s chemical properties, the resulting isotherms were cataloged as type I and type III [22,33,92]. The type I isotherms are observed for SiO_2_-C, Al_2_O_3_-C, and ZrO_2_-C nanoparticles and are characterized by the high affinity between the adsorbent and the adsorbate at low dosages and an adsorption limit uptake at high surfactant dosages [33,92]. Type III isotherms were observed for MgO-S, SiO_2_-S, and ZnO-S nanoparticles and are characterized by the generation of a multilayer adsorption process. Type III adsorption isotherms are related to weaker adsorbent–adsorbate interactions, which conduct a non-well-distributed positioning over the available surface area. Instead, adsorbed molecules are clustered around preferred surface positions, promoting adsorbate–adsorbate interactions [30,92]. According to the maximum adsorbed amount, type I and type III isotherm systems exhibit the following order: SiO_2_-C > Al_2_O_3_-C > ZrO_2_-C > MgO-S > SiO_2_-S > ZnO-S. The results show that the maximum adsorbed amount for fixed surfactant initial dosages below 5000 mg·L^−1^ decreases as follows: SiO_2_-C > Al_2_O_3_-C > ZrO_2_-C > MgO-S > SiO_2_-S > ZnO-S. As expected, the larger surface area of SiO_2_-C allows for a superior adsorption capacity. However, the chemical complexity and the role of the surface charges of the nanoparticles assessed explain why the adsorption behavior is not only connected to the available surface area [93,94,95].

The solid–liquid equilibrium (SLE) model was employed to describe surfactant adsorption on the nanoparticle’s surface, and their parameters are displayed in Table 4. H parameter defines the affinity between the functional groups on the surface of nanoparticles and the surfactant adsorbed [27,30,33]. Despite this, the H parameter fit to experimental results resulted in the following increasing values: SiO_2_-C < Al_2_O_3_-C < ZrO_2_-C < MgO-S < SiO_2_-S < ZnO-S, which implies the higher affinity degree toward surfactant with SiO_2_-C nanoparticles and lowest affinity degree for ZnO-S [33]. The experimental results and the SLE model show a greater reduction in surfactant adsorption in the rock surface for SiO_2_-C, Al_2_O_3_-C, and ZrO_2_-C for initial surfactant dosages below 5000 mg·L^−1^. In the case of the MgO-S, SiO_2_-S, and ZnO-S nanoparticles, the generation of weaker interactions with surfactants leads to higher adsorption on the rock surface.

### 3.3. Nanoparticle Selection

Nanoparticles were selected based on the capillary numbers and the clay inhibition capacity of the prepared CF in the presence of nanoparticles. The dimensionless capillary number was calculated using wettability and IFT values and is employed to consider the changes in fluid mobilization associated with viscous drag forces against surface tension forces across the interface generated by the multiphase flow at the reservoir [71]. Clay swelling inhibition was carried out through rheology tests to evaluate the treatment positive dual effect in well stimulation when there is a nanoparticle dosage in CF [17,30]. Ultimately, nanoparticles with a higher capillary number and a lower PV were selected according to Equations (6) and (7).

#### 3.3.1. Wettability Test

**Figure 5:**
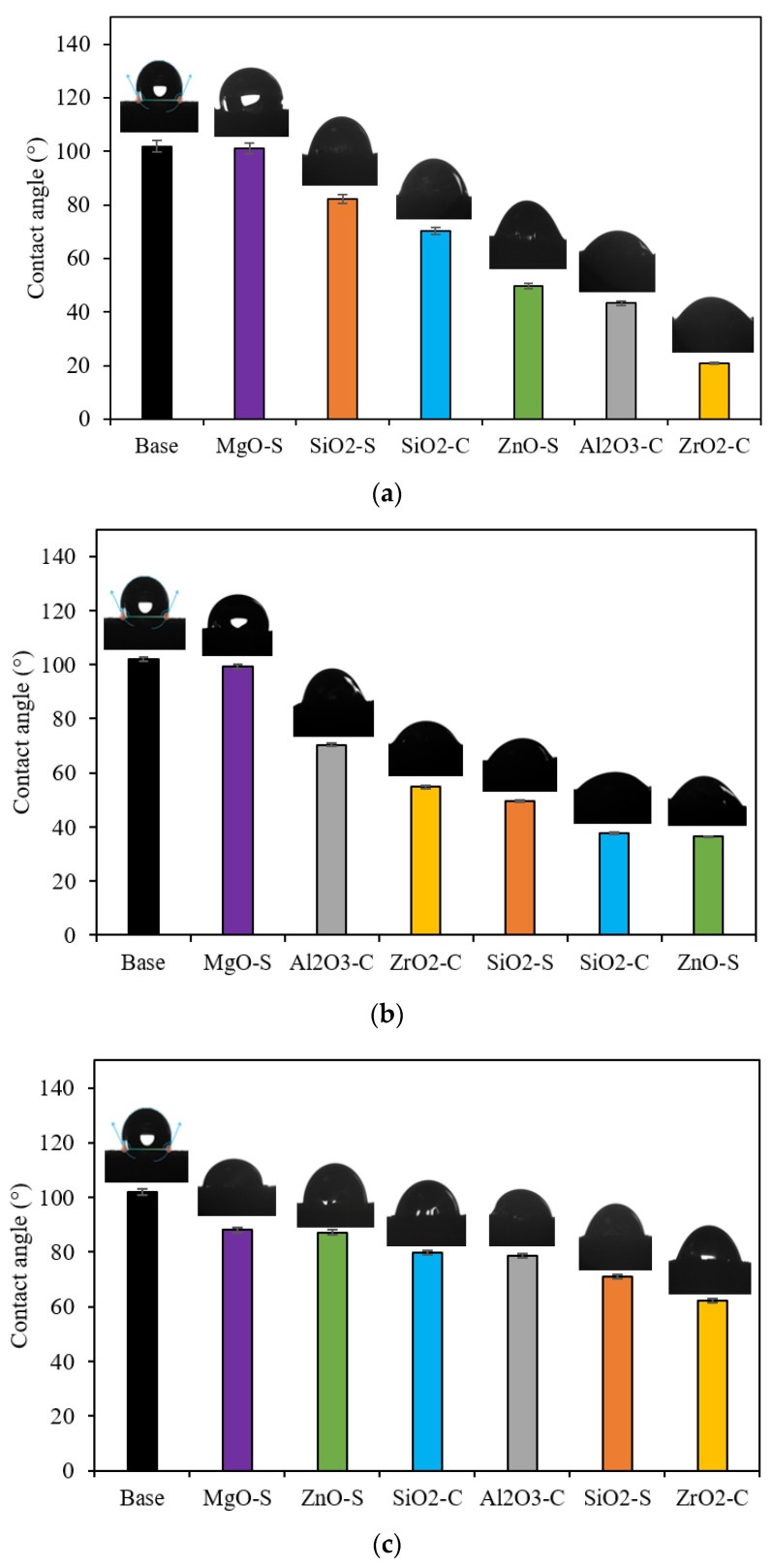
Contact angle values at 25 °C for sandstone surface treated with MgO-S, ZnO-S, SiO2-S, Al2O3-C, ZrO2-C, and SiO_2_-C for dosages of (**a**) 100 mg·L^−1^, (**b**) 300 mg·L^−1^, and (**c**) 500 mg·L^−1^.

Figure 5 presents the results of the wettability test carried out through contact angle measurements in oil-aging sandstone surfaces. First, the contact angle measurement was obtained over a non-treatment surface labeled as a base. Second, a test was conducted on an aging surface treated with a nanoparticle-free CF. The aged rock sample exhibits CA values of 135.2°. However, when CF is employed to restore the wettability, the CA reaches a value of 101.9° and yields a reduction of 24.6%. Although the CA value was reduced in the presence of the CF, oil-wettability systems are evident [96,97].

The addition of nanoparticles to CF shows contact angle reductions, favoring water-wet media for all nanoparticles, except in the MgO-S at nanoparticle concentrations lower than 300 mg·L^−1^. The alteration of the rock surface wettability implies a substitution of the habitual interface toward a hydrophilic one. This can be explained by the nanoparticle deposition onto the rock surface by disjoining pressure phenomena, which depends on the electrical charges and dispersive capability [30,98,99]. Nevertheless, wettability alteration reaches a limit in angle reduction. After this point, the increase in concentration has a less than optimal effect, which is associated with the nanoparticle aggregation phenomenon that changes molecular interactions between the nanoparticle surface, the surfactant, and the rock surface; these factors affect the distribution of nanoparticles onto the rock [100,101]. The nanomaterials exhibited the maximum capacity to promote a water-wet system at approximately 100 mg·L^−1^ for Al_2_O_3_-C and ZrO_2_-C, and at near 300 mg·L^−1^ for SiO_2_-S, SiO_2_-C, and ZnO-S. The greater reduction is achieved with ZrO_2_-C in 100 mg·L^−1^ dosages, resulting in a contact angle of 28.9°; this is a 78.6% reduction in the blank base case and a 71.6% reduction in the case of CF without nanoparticles.

Furthermore, the contact angles for the nanoparticles of ZrO_2_-C (100 mg·L^−1^), ZnO-S (300 mg·L^−1^), SiO_2_-C (300 mg·L^−1^), and ZnO-S (100 mg·L^−1^) are 28.9°, 36.4°, 37.8°, and 37.4°, respectively. These results show that the performance of these nanoparticles is influenced by the synergic effect between the adsorbed-surfactant and free-surfactant molecules, and its implication in dispersion degree, nanoparticle electric charges, and nanomaterial deposition [101,102,103]. The formation of a highly hydrophilic layer due to disjoining pressure that increases at the wedge zone for nanoparticle magnitude depends on the charges, size, and concentration of the nanoparticle [101,104].

#### 3.3.2. Interfacial Tension

**Figure 6:**
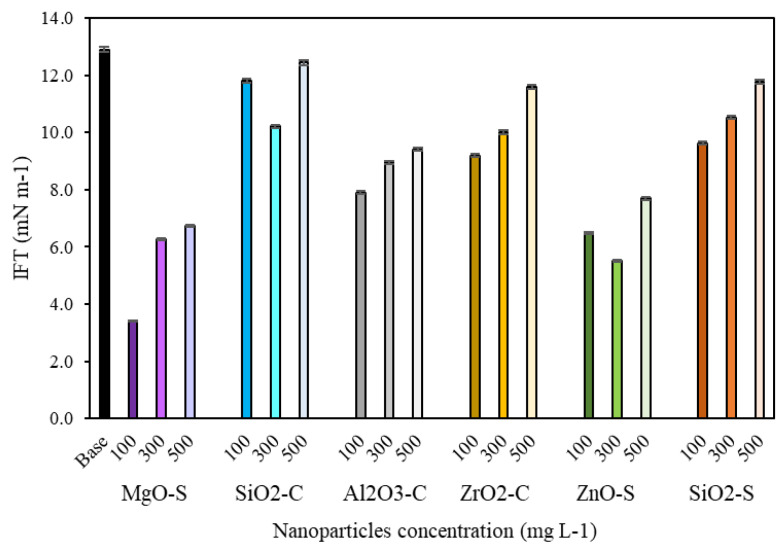
IFT values for measurements between crude oil and NanoCF-based MgO-S, ZnO-S, SiO_2_-S, SiO_2_-C, Al_2_O_3_-C, and ZrO_2_-C for 100, 300, and 500 mg·L^−1^.

IFT test results are presented in Figure 6. A better result of the IFT test was achieved with MgO-S at 100 mg·L^−1^ (2.5 mN·m^−1^), with an 80.4% reduction. However, one must note that ZnO-S nanoparticles with dosages from 100 to 500 mg·L^−1^ also promote important reductions between 40% and 72%, which minimize the resistance to flow and favor the oil mobilization in the multiphase reservoir condition [68]. In addition, according to the batch adsorption isotherms, MgO-S and ZnO-S correspond to nanoparticles with a lower affinity toward the surfactant, allowing the presence of more free-surfactant molecules than adsorbed-surfactant molecules. On the other hand, both nanoparticles exhibit a positive charge surface, which could endorse the free/adsorbed-surfactant molecules at the interface, enhancing the interfacial tension reduction [30,55,101]. Another noteworthy finding is that nanoparticles reach a limit in IFT reduction at a maximum dosage of between 100 and 300 mg·L^−1^. The nanoparticle concentration above 500 mg·L^−1^ in CF has a less than optimal effect after this point, which suggests an aggregation phenomenon that does not allow the adequate positioning of the surfactant along with the interface [101].

#### 3.3.3. Capillary Number (Nc)

**Figure 7:**
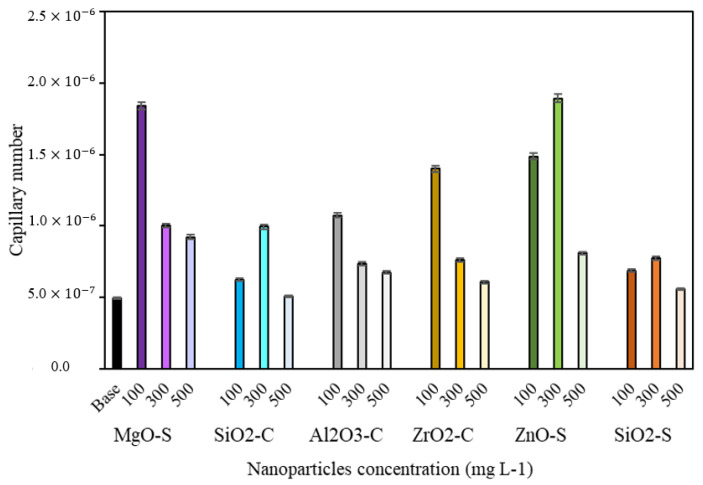
Capillary numbers for completion fluid (CF): base (black bar), MgO-S (purple bars), ZnO-S (green bars), ZrO_2_-C (yellow bars), Al_2_O_3_-C (grey bars), SiO_2_-S (orange bars), and SiO_2_-C (blue bars) at nanoparticle dosages of 100, 300, and 500 mg·L^−1^.

Figure 7 shows the capillary number of the CF systems in the absence and presence of nanoparticles. The average fluid velocity and the core sectional area were determined using the conventional injection fluid rate during the displacement test under laboratory conditions. The improvement in CF performance after adding MgO-S, ZnO-S, and ZrO_2_-C in dosages below 300 mg·L^−1^ is evident. The greater increments of capillary number are achieved with ZnO-S at 100 mg·L^−1^ and MgO-S at 100 mg·L^−1^ concentrations. These dosages generate an increase of 4.0 times the base. Other systems with exceptional qualities that improve crude mobility are ZnO-S in 300 mg·L^−1^, ZrO_2_-C in 100 mg·L^−1^, and Al_2_O_3_-C in 100 mg·L^−1^, with increases of more than 2.0 times the capillary number concerning CF in the absence of nanoparticles. The results show that nanomaterials with low surfactant affinity and, consequently, larger concentrations of free surfactants achieve an optimal equilibrium point to perform better by altering superficial and interfacial phenomena that regulate the flow on the porous medium. Finally, the reduction in IFT and contact angle values accomplished with the nanoparticles minimizes the resistance to fluid caused by tension between reservoir phases and improves the oil linear displacement efficiency by permitting the mobilization of immobile oil drops across pores [71].

**Figure 8:**
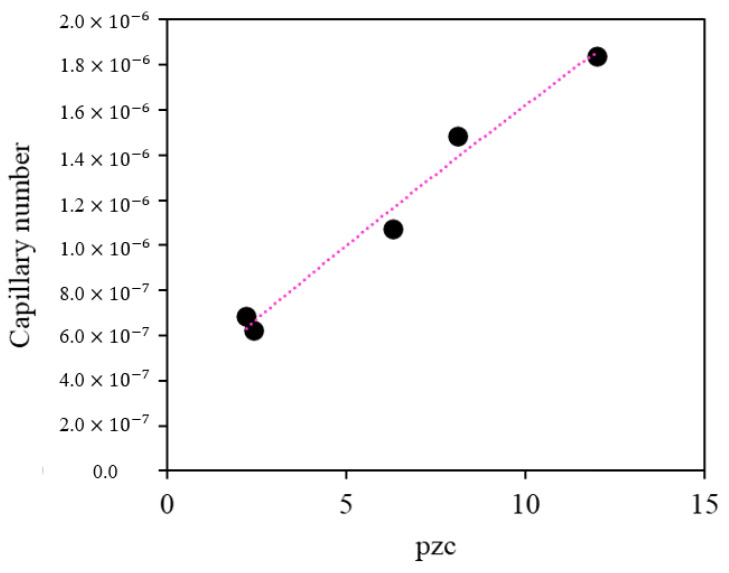
Relationship between the capillary number and the point of zero charge (pH_pzc_).

The results indicate that nanoparticles with a higher point of zero charge exhibit a greater increase in the capillary number, as shown in Figure 8. This is because the relationship between viscous and capillary forces was favored due to a significant decrease in interfacial tension in the presence of MgO and ZnO nanoparticles. For the evaluated system, these results suggest that the surfactant compound is efficiently delivered to the crude oil/brine interface in the presence of the positively charged nanoparticles.

#### 3.3.4. Clay Swelling Inhibition

**Figure 9:**
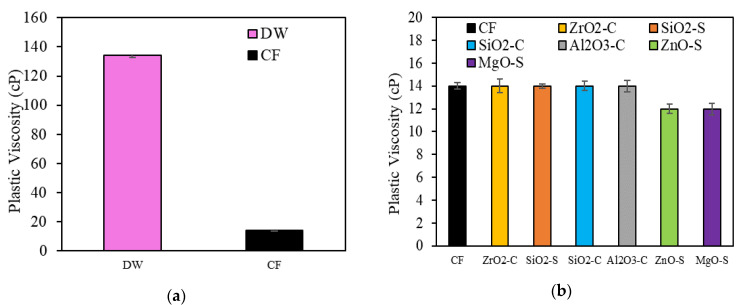
Clay swelling inhibition results through the rotational viscosimeter method for (**a**) deionized water (DW) and nanoparticle-free CF; and (**b**) nanoparticle-free CF and NanoCF.

Figure 9a presents clay swelling results. The reference system for the CF performance was obtained by adding natural bentonite at a 0.09 Kg·L^−1^ dosage to deionized water (DW), which has a PV value of 134 cp. The PV values of nanoparticle-free CF were compared to the NanoCF values. The CF without nanoparticles exhibits a decrease of 89.6% in PV, which suggests that the addition of KCl affects the clay swelling process; this effect is caused by cation exchange mechanisms between the brine charges and rock surface [105,106]. Figure 9b shows that using nanoparticles at 100 mg·L^−1^ in the CF accomplished an incremental swelling inhibition for the MgO-S and ZnO-S cases. This reduction is equal to 14.3% for both nanoparticles. Phenomenologically, it is associated with the nanoparticle positive charge interaction on the clay structure, which helps to increase electrostatic forces around the clay surface and counteract the tendency to swell; this prevents the dispersion of larger solids in the aqueous samples, which can increase the fluid PV [4,17].

The results show that nanoparticles with a pH_pzc_ above 6.1 (pH of CF) will be positively charged, which means that cation exchange will be favored. This is relevant during the dissociation of cations prevalent in the clay when it comes into contact with water. The negatively charged structural units can interact with the positive charges of the nanoparticles, whereby the repulsion between the clay particles and their swelling capacity is reduced [106].

The exceptional qualities of ZnO-S and MgO-S in the enhancement of the capillary number and the endorsement of the clay swelling inhibition suggest that the nanoparticles can be evaluated in a clay-rich reservoir to increase the mobility of the crude oil. ZrO_2_-C is also acceptable for enhancing crude oil mobility. Based on these results, ZnO-S, MgO-S, and ZrO2-C nanoparticles have been selected to evaluate the surface modification with CTAB to potentiate the performance of the nanoparticles for the inhibition of clay swelling.

### 3.4. Nanoparticle Surface Modification with CTAB

**Figure 10:**
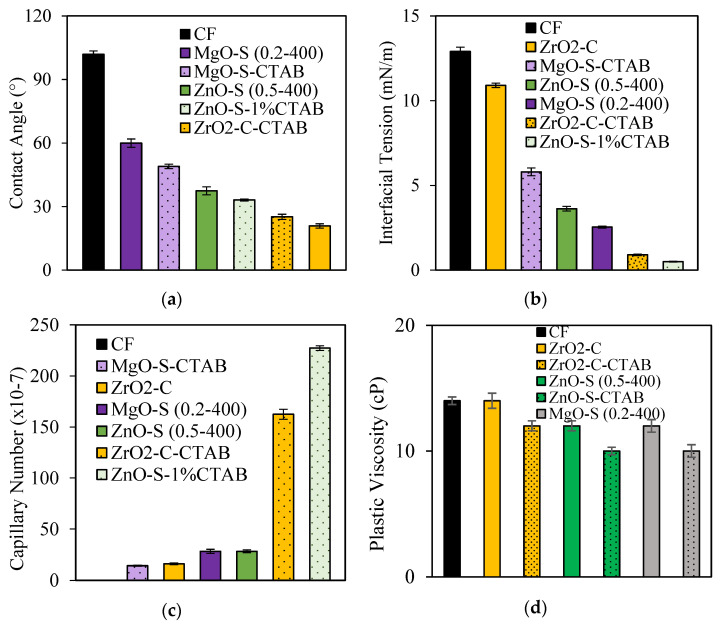
(**a**) Contact angles at 25 °C for sandstone treated with CF-containing surface- and non-surface-modified nanoparticles with CTAB. (**b**) Interfacial tension (IFT) values for measurements between crude oil and CF-containing surface- and non-surface-modified nanoparticles with CTAB. (**c**) Capillary number for CF-containing surface- and non-surface-modified nanoparticles with CTAB. (**d**) Clay swelling inhibition results through the rotational viscosimeter method for CF-containing surface- and non-surface-modified nanoparticles with CTAB. Nanoparticle concentration: 100 mg·L^−1^.

Surface modification was developed to take advantage of the swelling inhibiting effect of CTAB [48,49,50,51]. The test was conducted at 100 mg·L^−1^ due to the importance of IFT in reducing resistance to multiphase [71,105]. Figure 10 panels (a–d) show the results from the measurements of the interfacial tension, contact angle, capillary number estimation, and clay swelling inhibition test, respectively. The inclusion of CTAB over the nanoparticle surface alters both the surface and the interface interactions due to the presence of multiple adsorbed/free-tensoactive compounds in the CF formulation. For ZnO-S and ZrO_2_-C nanoparticles, the presence of CTAB potentiates the interfacial tension reduction with reductions of over 95% compared with the CF without nanoparticles. The nanoparticles display the following trend in promoting the IFT reduction: ZnO-S-CTAB > ZrO_2_-C-CTAB > MgO-S > ZnO-S > ZrO_2_-C. For contact angle measurements, all surface-modified nanoparticles promote more water-wet surfaces than the nanoparticles without surface modification. The positive effect of the CTAB in the enhancement of crude oil mobility was confirmed for the capillary number. The most effective nanoparticles are ZnO-S-CTAB, followed by ZrO_2_-C-CTAB, with an increase of over ten times the capillary number value of the CF without nanoparticles. Finally, regarding the clay swelling inhibition test, it was confirmed that the presence of CTAB reduces the PV by over 25% of the clay swelling for ZnO-S-CTAB and MgO-S-CTAB; this enhances the behavior in comparison with the nanoparticles without any surface modification.

### 3.5. Dynamic Test

**Figure 11:**
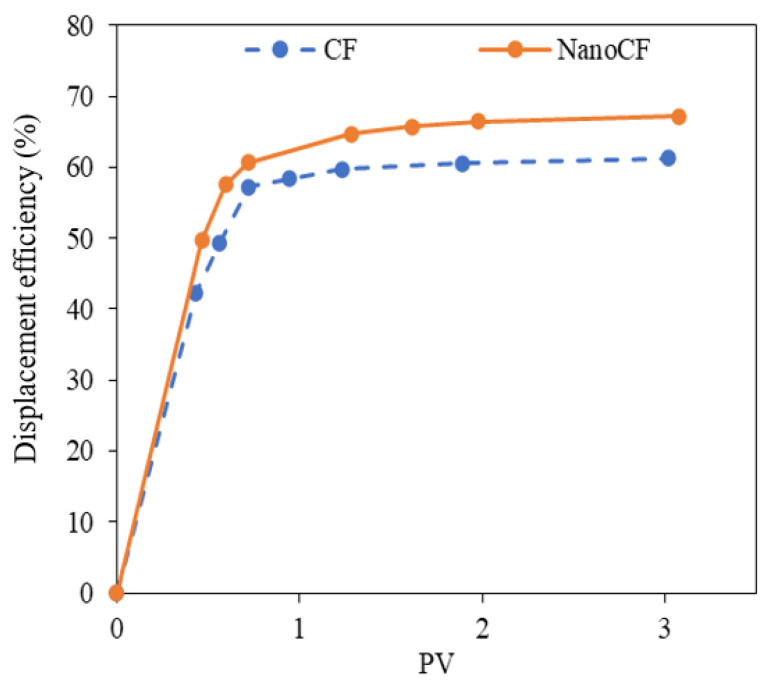
Oil linear displacement efficiency curves at atmospheric conditions for the base (blue symbols) and the selected NanoCF with ZnO-S-CTAB nanoparticles at 100 mg·L^−1^ (orange symbols).

Figure 11 displays the oil linear displacement efficiency curves for the base scenario and after the injection of NanoCF. A 5.9% increase in oil linear displacement efficiency occurred after soaking with the selected NanoCF. This behavior is associated with increased oil mobility due to increased capillary number. Furthermore, there was no evidence of clay swelling during the dynamic test. For this reason, the NanoCF composed of 100 mg·L^−1^ of ZnO-S-CTAB can potentially function as an agent that may be induced by the increase in the oil mobility, caused by IFT reduction, and may potentiate the clay swelling inhibition in the CF formulations.

## 4. Conclusions

Including oxide nanoparticles in commercial CF modifies the interactions at the surface/interface scale, promoting a reduction in interfacial tension and the generation of a water-wet surface in aged rock surfaces. Moreover, positive charge nanoparticles (MgO-S and ZnO-S) promote the adsorbed/free-surfactant interaction at the oil-and-water interface, showing the most promising results in increased crude oil mobility at the pore scale conditions and the inhibition of clay swelling. ZnO-S nanoparticles potentiated with surface modification using CTAB allow additional alterations in the fluid–fluid–rock interactions that endorse the dual positive effect in both targets. ZnO-S-CTAB at a concentration of 100 mg·L^−1^ in the completion fluid reduces interfacial tension between water and oil by up to 95% through the synergistic effect of binary surfactant mixtures and covered surface nanoparticles. In addition, the developed nanomaterials promote water-wettability systems and IFT reductions. That leads to an over tenfold increase in the capillary number of CF in the absence of nanomaterials. At dynamic conditions, the nanofluid injection treatment leads to a rise in the oil linear displacement efficiency of 5.9%. The incorporation of similar nanoparticles can facilitate completion fluid fulfilling the purpose of inhibiting the clay swelling and stimulating the well from the beginning of its productive life.

## Figures and Tables

**Table 1:** Properties of formation brine and commercial surfactant.

Fluid	Property				
	Density (g·mL^−1^)	pH	Salinity (g·L^−1^)	Conductivity (µS·cm^−1^)	Total Dissolved Solids (TDS) (mg·L^−1^)
Formation brine	1.02	6.5	35,380	1660.00	26,830.0
Commercial surfactant	0.99	4.3	885	54.75	813.9

**Table 2:** Petrophysical properties of the sandstone sample.

Property	Value
Length (cm)	8.1
Diameter (cm)	3.2
Porous Volume (mL)	25.1
Porosity (%)	38.5
Liquid injection flow (mL∙min^−1^)	0.2

**Table 3:** Hydrodynamic diameter (D50), pHpzc, and Brunauer–Emmett–Teller surface area (SBET) of Al_2_O_3_-C, ZrO_2_-C, MgO-S, ZnO-S, SiO_2_-C, and SiO_2_-S.

Nanoparticle	Hydrodynamic Diameter D50 (nm)	pHpzc	Surface Area SBET (m^2^∙g^−1^)
Al_2_O_3_-C	150.1	6.3	27.1
ZrO_2_-C	55.6	2.9	31.6
MgO-S	29.3	11.5	59.7
ZnO-S	100.0	8.1	20.1
SiO_2_-C	11.8	2.4	380.0
SiO_2_-S	83.1	2.2	23.0

**Table 4:** Estimated solid–liquid equilibrium (SLE) model parameters for surfactant adsorption onto nanoparticles at 25 °C.

Nanoparticles	*H* (mg·g^−1^)	*K* (g·g^−1^)	*N_ads_* (g·g^−1^)	*RSME* (%)
SiO_2_-C	1.1	3.3	0.6	5.4
Al_2_O_3_-C	6.6	10.1	0.4	2.9
ZrO_2_-C	15.3	13.5	0.4	3.8
MgO-S	37.9	12.5	0.9	7.5
SiO_2_-S	47.6	10.1	13.7	11.9
ZnO-S	55.4	11.0	19.2	7.4

## Data Availability

The data presented in this study are available on request from the corresponding author.
